# Association of Myocardial Energetic Efficiency with Circumferential and Longitudinal Left Ventricular Myocardial Function in Subjects with Increased Body Mass Index (the FATCOR Study)

**DOI:** 10.3390/jcm10081581

**Published:** 2021-04-08

**Authors:** Costantino Mancusi, Helga Midtbø, Nicola De Luca, Hilde Halland, Giovanni de Simone, Eva Gerdts

**Affiliations:** 1 Hypertension Research Center, Department of Advanced Biomedical Sciences, University Federico II of Naples, 80131 Naples, Italy; nideluca@unina.it (N.D.L.); simogi@unina.it (G.d.S.); 2 Department of Heart Disease, Haukeland University Hospital, 5021 Bergen, Norway; helga.bergljot.midtbo@helse-bergen.no (H.M.); hilde.halland@uib.no (H.H.); Eva.Gerdts@uib.no (E.G.); 3 Department of Clinical Science, University of Bergen, 5020 Bergen, Norway

**Keywords:** myocardial performance, triglycerides, global longitudinal strain, left ventricular mass

## Abstract

Lower myocardial mechanic-energetic efficiency (MEEi), expressed as stroke volume/heart rate ratio (SV/HR) in mL/s/g of the left ventricular (LV) mass, is associated with the incidence of heart failure in subjects with cardiometabolic disorders. We explored the association of MEEi with LV systolic circumferential and longitudinal myocardial function in 480 subjects with increased body mass index (BMI) without known cardiovascular disease (mean age 47 ± 9 years, 61% women, 63% obese, 74% with hypertension) participating in the fat-associated cardiovascular dysfunction (FATCOR) study. Insulin resistance was assessed by the homeostasis model assessment insulin-resistance index (HOMA-IR). SV was calculated by Doppler echocardiography. The LV systolic circumferential myocardial function was evaluated by midwall fractional shortening (MFS) and longitudinal function by global longitudinal strain (GLS). Patients were grouped into MEEi quartiles. The lowest MEEi quartile (<0.41 mL/s per g) was considered low MEEi. The association of MEEi with MFS and GLS were tested in multivariable linear regression analyses. Patients with low MEEi were more frequently men, with obesity and hypertension, dyslipidemia and higher HOMA-IR index (all *p* for trend <0.05). In multivariable analyses, lower MEEi was associated with lower LV myocardial function by MFS and GLS independent of higher LV mass and clinical variables, including older age, male sex, presence of hypertension and a higher triglycerides level (all *p* < 0.05). In conclusion, in subjects with increased BMI without known cardiovascular disease participating in the FATCOR study, reduced MEEi was associated with lower LV myocardial function both in the circumferential and longitudinal direction, independent of cardiometabolic factors.

## 1. Introduction

Obesity is linked to the full spectrum of cardiac dysfunction, from subclinical left ventricular (LV) diastolic dysfunction to decompensated systolic heart failure [1]. Cardiac metabolism in obese individuals is characterized by a less efficient adenosine triphosphate (ATP) production and utilization, producing functional consequences that are linked to the increased prevalence of heart failure [2,3]. The switch in cardiac metabolic substrate in obese subjects from glucose to triglycerides may impair myocardial energy production and promote subclinical systolic dysfunction. Cardiac triglyceride content quantified by proton magnetic resonance spectroscopy was increased in subjects with impaired glucose tolerance or obesity and reduced systolic function [4]. Thus, assessment of myocardial metabolism and its efficiency may improve the understanding of cardiac involvement in obesity. Positron emission tomography (PET) can be used to measure myocardial energetics and efficiency, but this method is less available in large studies [5]. We generated a simple echocardiographic method for the estimation of myocardial energetic efficiency (MEE) as a ratio of myocardial external work to myocardial oxygen consumption, normalized per gram of LV mass (MEEi), suitable for the assessment of cardiac efficiency in clinical practice [6,7]. Low MEEi has been associated with obesity, type 2 diabetes mellitus, and with insulin resistance in non-diabetic patients [8,9]. Furthermore, it has been validated as a marker of increased cardiovascular (CV) event’s risk in patients with arterial hypertension [6] and in particular with the incidence of heart failure in the community-based Strong Heart Study [10]. The relation of MEEi to LV systolic myocardial function has not been yet evaluated. Thus, the aim of the present study was to explore the association of MEEi with parameters of LV systolic function in adults with increased BMI and free from CV disease.

## 2. Materials and Methods

The current study used data from the fat-associated cardiovascular dysfunction (FATCOR) study. The characteristics of the study population have been previously reported [11]. A total of 620 women and men aged 30 to 65 years with a BMI > 27.0 kg/m^2^ free from clinical cardiovascular disease were enrolled from 2009 to 2017 at Haukeland University Hospital, Bergen, Norway. The exclusion criteria were previous myocardial infarction, gastrointestinal disorder, severe psychiatric illness or the inability to communicate in the Norwegian language. The FATCOR study was conducted in accordance with the Declaration of Helsinki and with approval from the Regional Ethics Committee. Written informed consent was obtained from all participants. A total of 127 participants were excluded due to incomplete echocardiographic data (*n* = 51), insufficient echocardiographic image quality (*n* = 49) or hardware mismatch for the analysis of global longitudinal strain (GLS, *n* = 25), missing information on Doppler stroke volume (SV, *n* = 13) or withdrawal of informed consent (*n* = 2). All participants completed a standardized questionnaire reporting their medical history and use of any medication. Clinic blood pressure (BP) was measured in accordance with guidelines using an Omron M4 sphygmomanometer (Omron Healthcare Co. Ltd., Hoofdorp, The Netherlands) with an appropriately sized cuff [12]. The pulse pressure was calculated as the difference between clinic systolic and diastolic BP. A Diasys Integra II apparatus (Novacor, Cedex, France) was used for 24-h ambulatory blood pressure monitoring. An average 24-h systolic BP ≥130 mmHg and/or 24-h diastolic BP ≥80 mmHg was considered elevated. Hypertension was considered present if the 24-h ambulatory BP was elevated or the participants reported the use of antihypertensive medication [12]. Obesity was defined as BMI 30.0 kg/m^2^. In accordance with the criteria from the American Diabetes Association [13], diabetes mellitus was considered present if fasting blood glucose ≥7 mmol/L, 2-h blood glucose ≥11.1 mmol/L after a 75-g oral glucose test, or a glycated hemoglobin A1c ≥6.5%. Insulin resistance was assessed by the homeostasis model assessment insulin-resistance index (HOMA-IR). The estimated glomerular filtration rate was calculated using the Chronic Kidney Disease Epidemiology Collaboration equation. Echocardiography was performed with a GE Vivid E9 scanner (GE Vingmed Ultrasound, Horten, Norway) following a standardized imaging protocol [11]. The images were analyzed using Image Arena software version 4.4 (TomTec Imaging Systems GmbH, Unterschleissheim, Germany) in the Echocardiography Core Laboratory at the University of Bergen, Bergen, Norway. All images analyzed were quality assured by proofreading by a single expert reader (EG). The current American Society of Echocardiography and European Association of Cardiovascular Imaging guidelines for chamber quantification were applied for quantitative echocardiography [14]. LV mass was indexed for height^2.7^ as recommended in obesity [12]. LV hypertrophy was defined by sex-specific cut-off values for LV mass/height^2.7^ (>46.7 g/m^2.7^ in women and >49.2 g/m^2.7^ in men). The relative wall thickness (RWT) was calculated from the posterior wall thickness/LV internal radius ratio and considered increased if ≥0.43 (concentric LV geometry). The SV was calculated by PW-Doppler at the aortic valve hinging points. Midwall fractional shortening (MFS) was calculated using a previously reported formula [15]. To estimate MEE, myocardial oxygen consumption (MVO2) was estimated by the “double product” heart rate (HR) times brachial systolic BP (SBP). Stroke work (SW) was estimated by SBP × SV. Accordingly, MEE per heartbeat was the ratio between the SW and MVO2:

(1) $$\mathrm{MEE}\;=\;\frac{\mathrm{SW}}{\mathrm{MVO}2}=\frac{\mathrm{SBP}\times \mathrm{SV}}{\mathrm{SBP}\times \mathrm{HR}}=\frac{\mathrm{SV}}{\mathrm{HR}/60}$$

Thus, MEE could be measured as the ideal amount of blood pumped by one single heartbeat in one second. Since this quantity is related to the amount of myocardium available for pump performance [7], MEE was normalized for LV mass (MEEi) for estimating the theoretical quantity of blood pumped by each gram of LV mass in one second. Speckle tracking echocardiography analysis of LV peak systolic longitudinal strain was done offline on a workstation equipped with EchoPac BT 202 (GE Vingmed Ultrasound, Horten, Norway) with excellent reproducibility [16]. The peak systolic longitudinal strain was assessed in the apical two-, three- and four-chamber views using the automated imaging function. The endocardial border was traced automatically, and the end-systole was defined by aortic valve closure. After software processing, the quality of tracking was assessed visually, and if the tracking was poor, the segment was excluded. The global longitudinal strain (GLS) was calculated as the average peak systolic longitudinal strain in the 17 LV segments [17].

Data were analyzed using SPSS (version 21.0; SPSS, Chicago, IL, USA). The study cohort was divided into MEEi quartiles. The lowest MEEi quartile was considered as low MEEi (<0.41 mL/s/g). Analysis of variance was used to compare continuous variables among MEEi quartiles, using polynomial linear contrast for trend analysis. The χ^2^ distribution was used to compare categorical variables and Kendall’s t–b was used for trend analysis. Univariable and multivariable regression analyses were used to identify the association of MEEi with circumferential and longitudinal LV myocardial systolic function (MFS and GLS) after adjusting for age, sex, BMI, the presence of hypertension, HOMA index, serum triglycerides, LV mass and concentric geometry. The odds ratio (OR) and associated 95% confidence intervals (CI) were reported. A *p*-value of <0.05 was considered statistically significant. All variables used in the multivariable models were analyzed for multicollinearity, by computing the linear variance inflation factor (VIF). VIF was always less than 1.5.

## 3. Results

A total of 480 patients were included in the analysis (mean age 47 ± 9 years, 61% women, 63% obese, 74% hypertensive). The lower MEEi quartile was confirmed to be associated with a greater proportion of men, as well as obesity and hypertension (all *p* for trend <0.05). The lower MEEi quartile was also associated with a worse lipid profile and higher HOMA-IR index (all *p* for trend <0.05) (Table 1). Ejection fraction, MFS and GLS decreased and LV mass and concentric geometry increased in parallel with the lower MEEi quartile (Table 2).

**Figure 1:**
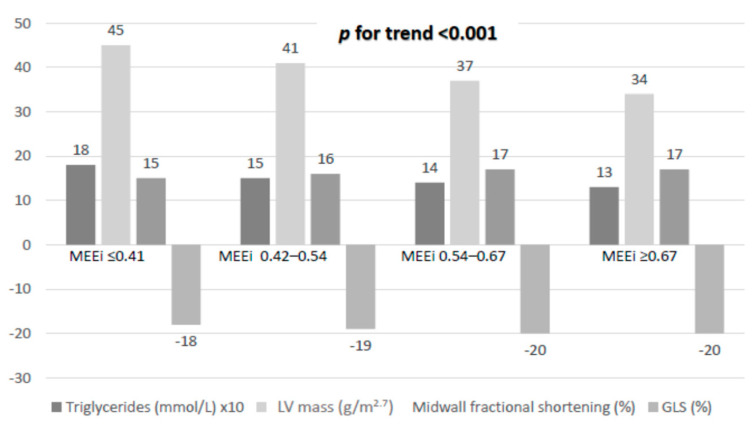
Levels of triglycerides, LV mass, midwall fractional shortening and global longitudinal strain among quartiles of MMEi.

In univariable analyses, the lower MEEi quartile was paralleled by increasing serum triglycerides level and LV mass, and by lower LV systolic myocardial function assessed by MFS and GLS (Figure 1).

In multivariable analysis, lower MEEi was independently associated with older age, male sex, the presence of hypertension and an increased triglycerides level (all *p* < 0.05). After adjusting for these factors as well as for LV geometry, lower MEEi remained independently associated with lower LV myocardial function by MFS and GLS (all *p* < 0.05) (Table 3).

## 4. Discussion

The present study advances our understanding of the association of obesity with heart failure. Our study demonstrates that LV systolic function, assessed by MFS and GLS, is independently related to MEEi among adults with increased BMI and free from CV disease in parallel. Low MEEi also parallels worse metabolic profile and higher prevalence of abnormal LV geometry, as also shown in previous studies in different populations, expanding previous results on metabolic and hemodynamic correlates of reduced myocardial efficiency [6,8].

Reduced GLS has been reported in arterial hypertension, in particular when obesity or LV hypertrophy is present [18]. Increased myocardial fat accumulation and interstitial myocardial fibrosis are associated with a reduction in GLS, [19] which in turn affects CV prognosis [20,21]. In line with these previous observations, lower MEEi was paralleled by lower GLS in the present study.

In obesity, an excess in circulating triglycerides is associated with increased fat storage in the epicardium, fat infiltration in the myocardium and subsequent impaired myocardial function [22]. In the obese heart, energy is mostly produced through the oxidation of free fatty acids (FFA) [23], a process requiring higher MVO2 than glucose-pyruvate oxidation with a reduced ratio between produced ATP and MVO2 [24]. The obese heart also loses the ability to switch substrates between FFA and glucose in cases of high glucose availability or oxygen deficiency, which is postulated to lead to reduced myocardial efficiency. In a study using cardiac magnetic resonance in overweight subjects, elevated myocardial triglyceride content was associated with increased LV mass and reduced LV systolic function, factors that were both associated with lower MEEi in the present echocardiographic study [25,26]. Visceral fat, including epicardial fat, promotes alterations in myocardial structure and function and subsequent development of heart failure [27]. Recently, Losi et al. demonstrated that low MEEi was a predictor of incident heart failure not related to previous acute myocardial infarction, in subjects with initially normal LV ejection fraction participants to the Strong Heart Study [10].

Our results demonstrate that in some patients with increased BMI but free from CV disease, at a given level of LV mass, the left ventricle works inefficiently with high energy-wasting, a condition associated with high CV risk phenotype. We demonstrate that systolic myocardial dysfunction is also part of this adverse cardiometabolic phenotype. Interestingly, low MEE is associated with both reduced MFS and GLS, strongly suggesting that both longitudinally and circumferentially, dysfunction contributes to determining reduced efficiency in obese/overweight individuals as previously reported [28].

Compared to the directly invasive PET measures of myocardial metabolism, our indirect approach is more feasible in larger studies and potentially clinically applicable to the daily care of patients. This approach has a strong rationale. The calculation of stroke work has been invasively validated and largely adopted [29], and should be considered well representative of LV external systolic work. The double product of heart rate by systolic pressure is a potent and reliable predictor of MVO2 [30], especially in steady-state conditions [31] and validated in animal experiments [32,33]. Our method for the estimation of MEEi, despite being less accurate than PET methods, is a reliable estimation of myocardial efficiency that can be used in patients with no more than mild valvular heart disease, during routine clinical practice.

Due to the cross sectional dataset available in our study, the prognostic impact of MEEi cannot be assessed in this particular subset of patients. Future prospective studies are needed in obese/overweight subjects without prevalent CV disease.

## 5. Conclusions

In conclusion, our study demonstrates that reduced MEEi in subjects with increased BMI is associated with lower LV myocardial function both in the circumferential and longitudinal direction, independent of cardio metabolic factors even in subjects without prevalent CV disease. An increased serum level of triglycerides was a main metabolic characteristic of reduced MEEi.

## Figures and Tables

**Table 1:** Clinical characteristics and metabolic risk profile in subjects grouped in quartiles of MEEi (mL/s/g).

	MEEi ≤ 0.41 (*n* = 111)	MEEi 0.42–0.54 (*n* = 120)	MEEi 0.54–0.67 (*n* = 125)	MEEi ≥ 0.67 (*n* = 124)	*p* for Trend
Age (years)	48 ± 9	49 ± 9	46 ± 9	47 ± 9	0.021
Women	42%	58%	68%	75%	<0.001
BMI (kg/m^2^)	33 ± 5	32 ± 4	32 ± 4	31 ± 4	<0.001
Fat Free mass (%)	65 ± 13	60 ± 13	58 ± 11	56 ± 10	<0.001
Hypertension	85%	76%	71%	65%	<0.001
Obesity	75%	62%	67%	48%	<0.001
Diabetes	10%	9%	4%	7.5%	0.146
Systolic BP (mmHg)	135 ± 16	132 ± 17	126 ± 14	125 ± 15	<0.001
Diastolic BP (mmHg)	86 ± 9	84 ± 10	80 ± 8	79 ± 8	<0.001
Heart rate (bpm)	72 ± 11	70 ± 10	67 ± 10	63 ± 9	<0.001
Fasting plasma glucose (mmol/L)	5.05 ± 0.5	5 ± 0.4	5 ± 0.5	4.8 ± 0.4	0.005
eGFR (ml/min/1.73m^2^)	94.7 ± 14	95.5 ± 13	98.6 ± 13	96.1 ± 13	0.106
Total cholesterol (mmol/L)	5.5 ± 1.2	5.5 ± 1	5.3 ± 1.1	5.5 ± 0.9	0.243
HDL-cholesterol (mmol/L)	1.2 ± 0.3	1.3 ± 0.4	1.3 ± 0.3	1.4 ± 0.3	0.013
Triglycerides (mmol/L)	1.8 ± 1.5	1.5 ± 0.8	1.4 ± 0.9	1.3 ± 0.6	<0.001
HOMA-IR	4.3 ± 6.5	3.8 ± 4.9	3.4 ± 4.7	2.8 ± 2.1	0.019

**Table 2:** Echocardiographic findings among subjects grouped in quartiles of MEEi (mL/s/g).

	MEEi ≤ 0.41 (*n* = 111)	MEEi 0.42–0.54 (*n* = 120)	MEEi 0.54–0.67 (*n* = 125)	MEEi ≥ 0.67 (*n* = 124)	*p* for Trend
LV end diastolic diameter (mm)	50 ± 5	50 ± 4	49 ± 5	48 ± 4	<0.001
Ejection fraction (%)	61 ± 6	62 ± 7	64 ± 6	63 ± 6	<0.001
Midwall fractional shortening (%)	15 ± 2	16 ± 3	17 ± 2	17 ± 2	<0.001
E/A	1.1 ± 0.32	1.1 ± 0.25	1.25 ± 0.36	1.29 ± 0.33	<0.001
LV mass (g/m^2.7^)	45 ± 11	41 ± 7	37 ± 7	34 ± 6	<0.001
Relative wall thickness	0.37 ± 0.08	0.36 ± 0.08	0.32 ± 0.07	0.31 ± 0.06	<0.001
LV hypertrophy	34%	17%	10%	2%	<0.001
Concentric geometry	23%	20%	10%	5%	<0.001
GLS (%)	−18 ± 3	−19 ± 3	−20 ± 3	−20 ± 3	<0.001

LV, left ventricular; GLS, global longitudinal strain.

**Table 3:** Covariables of MEEi in uni- and multivariable linear regression analyses.

	Univariate		Model 1 (R^2^ = 0.14)		Model 2 (R^2^ = 0.31)	
	Beta	*p*	Beta	*p*	Beta	*p*
Age (years)	−0.086	0.058	−0.092	0.991	0.001	0.991
Male sex	−0.240	0.0001	−0.234	0.0001	−0.059	0.130
BMI (kg/m^2^)	−0.174	0.0001	−0.181	0.0001	−0.068	0.173
Hypertension (n/y)	−0.189	0.0001	−0.098	0.038	−0.037	0.393
HOMA IR	−0.130	0.005	−0.054	0.238	−0.065	0.121
Triglycerides (mmol/L)	−0.167	0.0001	−0.089	0.05	−0.085	0.04
LV mass (g/m^2.7^)	−0.492	0.0001	–	–	−0.392	0.0001
Concentric geometry (n/y)	−0.204	0.0001	–	–	0.034	0.481
MFS (%)	0.322	0.0001	–	–	0.128	0.01
GLS (%)	−0.231	0.0001	–	–	−0.129	0.003

BMI, body mass index; HOMA-IR, homeostasis model assessment-insulin resistance index; LV, left ventricle; MFS, midwall fractional shortening; GLS, global longitudinal strain.

## Data Availability

The data presented in this study are available on request from the corresponding author. The data are not publicly available due to ethical restriction.
